# Targeting Gut Microbiota in Cancer Cachexia: Towards New Treatment Options

**DOI:** 10.3390/ijms24031849

**Published:** 2023-01-17

**Authors:** Concetta Panebianco, Annacandida Villani, Adele Potenza, Enrica Favaro, Concetta Finocchiaro, Francesco Perri, Valerio Pazienza

**Affiliations:** 1 Division of Gastroenterology, Fondazione IRCCS Casa Sollievo della Sofferenza, Hospital, Viale dei Cappuccini, 1, 71013 San Giovanni Rotondo, Italy; 2 Dietetic and Clinical Nutrition Unit, Fondazione IRCCS Casa Sollievo della Sofferenza, Viale dei Cappuccini, 1, 71013 San Giovanni Rotondo, Italy; 3 Department of Medical Science, University of Turin, 10124 Turin, Italy; 4 Department of Clinical Nutrition, Città della Salute e della Scienza, 10126 Turin, Italy

**Keywords:** cancer cachexia, gut microbiota, microbiota manipulation strategies

## Abstract

Cancer cachexia is a complex multifactorial syndrome whose hallmarks are weight loss due to the wasting of muscle tissue with or without the loss of adipose tissue, anorexia, systemic inflammation, and multi-organ metabolic alterations, which negatively impact patients’ response to anticancer treatments, quality of life, and overall survival. Despite its clinical relevance, cancer cachexia often remains an underestimated complication due to the lack of rigorous diagnostic and therapeutic pathways. A number of studies have shown alterations in gut microbiota diversity and composition in association with cancer cachexia markers and symptoms, thus supporting a central role for dysbiosis in the pathogenesis of this syndrome. Different tools of microbiota manipulation, including probiotics, prebiotics, synbiotics, and fecal microbiota transplantation, have been investigated, demonstrating encouraging improvements in cachexia outcomes. Albeit pioneering, these studies pave the way for future research with the aim of exploring the role of gut microbiota in cancer cachexia more deeply and setting up effective microbiota-targeting interventions to be translated into clinical practice.

## 1. Definition and Clinical Presentation of Cancer Cachexia

Cancer cachexia is a severe disorder occurring in a high percentage of cancer patients, whose prevalence varies for different types of tumor, with the highest incidence (up to 80%) observed in patients with pancreatic or gastric cancers, followed by lung, prostate, colon, breast cancers, and certain types of lymphomas and leukemias [1,2]. Cachexia is a multifactorial syndrome that clinically presents with weight loss due to the wasting of muscle tissue with or without the loss of adipose tissue, anorexia, inflammation, and metabolic alterations, which affect a patient’s quality of life, compliance, and response to therapies and overall survival [1,3,4,5]. Patients with cancer cachexia, indeed, experience worse clinical outcomes, also because weight loss requires dose reduction or the discontinuation of chemotherapy [6,7]. It is estimated that up to 20% of cancer patients die from cachexia [1], which compromises respiratory and cardiac muscle function [8].

According to the latest international consensus, the so-called Fearon criteria, cachexia is diagnosed when the patient presents an involuntary weight loss greater than 5% of the pre-illness body weight in the previous 6 months; or a decrease greater than 2% with a body mass index (BMI) less than 20, or a decrease greater than 2% in the presence of sarcopenia [4]. Actually, it is recognized that cachexia development progresses by steps in which cachexia, as above defined, can be preceded by a pre-cachexia stage, characterized by weight loss of less than 5%, anorexia, and metabolic changes, and possibly, it can be followed by refractory cachexia, in which the patient has a low performance status, is unresponsive to anticancer treatments, and has a life expectancy of fewer than 3 months [4].

**Figure 1:**
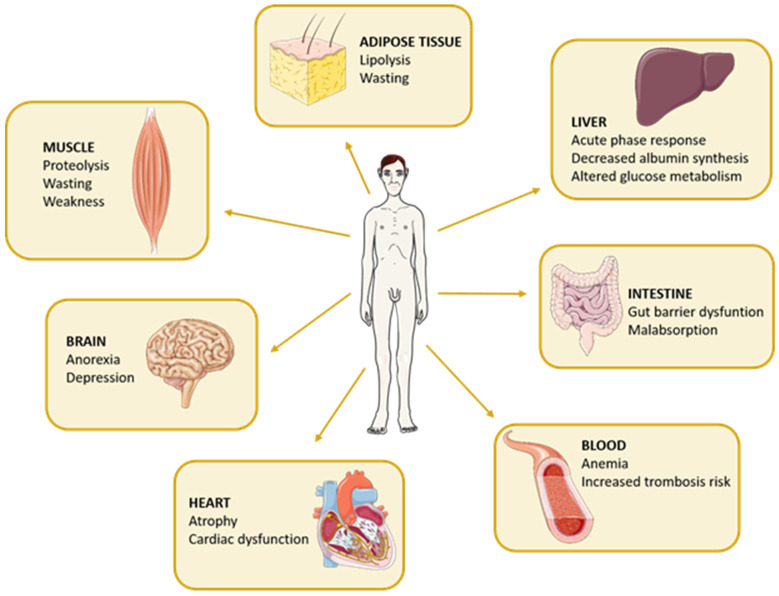
Multi-organ involvement in cancer cachexia. Apart from the wasting of muscle and adipose tissues, cancer cachexia causes functional alterations in many other organs such as in the liver, intestine, blood circulation, heart, and brain.

The pathogenesis of cachexia syndrome depends on a state of chronic systemic inflammation, which is fueled by both tumor and host cells, secreting pro-inflammatory cytokines such as TNF-α, IL-6, and IL-1 [3,8]. Although the muscle tissue is the leading target of such cytokines, cachexia can be considered a multi-organ syndrome since biochemical and functional alterations occur in many other body districts, such as adipose tissue (in which lipolysis occurs), the liver (with an increase in acute phase response, decrease in albumin synthesis, and altered glucose metabolism), gastrointestinal tract (with malabsorption and disruption of the gut barrier), blood (with anemia and increased thrombosis risk), brain (with anorexia and depression) and heart (with atrophy and cardiac dysfunction), as represented in Figure 1 and exhaustively reviewed elsewhere [1,5,8,9]. 

Apart from weight loss and fatigue, cachectic patients often clinically present with high levels of C reactive protein (CRP) and low levels of albumin as a consequence of inflammation [1], but so far, specific biomarkers that could help the early detection of cancer cachexia are lacking [9]. Despite its clinical relevance, cancer cachexia often remains a neglected medical problem for which a valid diagnostic approach and an effective therapeutic strategy are lacking. 

## 2. Emerging Treatment Options of Cancer Cachexia

The body wasting that occurs in cancer cachexia has long been considered a nutritional syndrome caused by anorexia and malnutrition [10]. It is now clear that the negative protein and energy balance characterizing cachexia results from a combination of decreased energy intake and increased energy expenditure [1,2], the latter due to a hypercatabolic state sustained by chronic inflammation [11]. For this reason, interventions based on nutrition support alone prove insufficient in counteracting cachexia [2,12]. A number of other strategies for treating cancer cachexia, which can be classified into different kinds of approaches, have been trialed or are currently under investigation. Among these, nutraceutical supplements (such as the Ω3 eicosapentaenoic acid and β-hydroxy-β-methylbutyrate), appetite stimulants (including the progestin megestrol acetate and the ghrelin receptor agonist anamorelin), anti-inflammatory drugs (such as corticosteroids, non-steroidal anti-inflammatory drugs, and cytokine modulators), metabolism-targeting drugs with pro-anabolic and/or anti-catabolic effects (e.g., the selective androgen receptor modulator enobosarm or the β-blocker espindolol), and physical exercise can be listed [9,13,14,15]. More recently, clinical trials on other new investigational products have started. Among these, Ponsegromab, a monoclonal antibody against the growth differentiation factor 15 (GDF-15), is currently under a Phase 2 investigation (NCT05546476), whereas the melanocortin-4 receptor antagonistic peptide TCMB07 (NCT05529849), the non-steroidal anti-inflammatory drug Ketorolac tromethamine (NCT05336266), and the JAK kinase inhibitor Ruxolitinib (NCT04906746) are in Phase 1 studies. Many of the aforementioned options, despite being successful at reaching some primary goals, such as increasing body weight or stimulating appetite, failed to fully reverse the complex alterations underlying cachexia syndrome, including increasing lean body mass, muscle function, and performance, improving quality of life, decreasing circulating inflammatory cytokines, and extending survival time [13]. For some approaches, adverse effects were found to outweigh beneficial ones [14]. As a result, to date, no universally approved molecule with a therapeutic indication for cancer cachexia exists, except for megestrol acetate, which has been approved in many countries around the world [9], including Europe [16], although its contribution in weight gain mainly relies on increasing adipose tissue and body fluids [17]. In light of all these observations, multimodal approaches to cancer cachexia are expected to provide better results than monomodal ones by targeting different aspects of the syndrome [14,18] and certainly, finding out novel and more effective interventions still represents a challenging goal to be pursued. For this to happen, a deeper understanding of the molecular basis of the immune-metabolic alterations characterizing cancer cachexia is required, in order to set up interventions targeted to pathogenetic mechanisms. 

## 3. Linking Gut Microbiota to Cancer Cachexia

### 3.1. Putative Microbial Mechanisms Contributing to Cachexia Development

Several lines of evidence suggest a role for intestinal microbiota in sustaining the inflammation and the metabolic alterations underlying cancer cachexia, as already demonstrated for cancer itself, for metabolic diseases such as obesity, diabetes, or cardiovascular diseases [19], and for other conditions that share clinical manifestations with cachexia, such as anorexia [20] and sarcopenia [21]. Countless mechanisms could explain the contribution of microbiota in the pathogenesis of cachexia. First, among all these, microbiota plays a fundamental role in maintaining gut barrier function [22] and dysbiotic states and, by increasing intestinal permeability, might put into blood circulation endotoxins and bacterial products [23,24] while also demonstrating a possibility of causing bacterial translocation from the intestinal lumen to the lamina propria, with the subsequent activation of host immune inflammatory response [24]. The composition of gut microbiota can affect the nutrient’s availability for host tissues, with particular reference to branched-chain amino acids (BCAA), which can be consumed by certain bacteria and, thus, are subtracted to muscle anabolic processes [25,26,27]. Consistently, lower plasma levels of BCAA were described in cachectic lung cancer patients compared to their non-cachectic counterparts [28]. Moreover, the microbiota composition also influences the production of certain bacterial metabolites, such as short-chain fatty acids (SCFAs), which own anti-inflammatory properties and have been found in a lower abundance in both cachectic mice [29] and patients [30]. In addition, gut bacteria, through the interaction with the enteric nervous system and the production of some metabolites with anorexigenic effects, such as SCFAs or bile acids, could also play a role in the reduction of appetite which is a typical feature of cachexia [25,31]. Finally, an interesting study showed that gut microbiota regulates skeletal muscle mass and function since germ-free mice displayed lower muscle mass, markers of muscle atrophy, and altered muscle metabolic functions, which were, however, restored after germ-free animals received microbiota transplantation [32]. Similarly, gut microbiota depletion by means of antibiotics induced skeletal muscle atrophy in mice through an altered bile acid metabolism [33]. 

### 3.2. Cachexia-Related Microbiota Profiles in Animal Models

A number of animal studies have demonstrated how shifts in microbiota compositions occur in different models of cancer cachexia. Bindels et al. set up a model of acute leukemia by injecting BCR-ABL transfected Ba/F3 lymphocytes in mice, which developed typical signs of cachexia, such as anorexia, inflammation, muscle wasting, and fat loss. These manifestations were accompanied by changes in gut microbiota with respect to the control mice. Indeed, although the number of total bacteria in the caecal content was comparable between the two animal groups, an overall decrease in *Lactobacillus* species was observed in leukemic mice, with a specific reduction in *L. reuteri* and *L. johnsonii/gasseri*, which were found to negatively correlate with the expression of atrophy markers in the muscle [34]. In a later study by the same authors, a deeper characterization of caecal microbiota was obtained through next-generation sequencing either in Ba/F3 leukemic or in C26 colorectal cancer mice models of cachexia [35]. In both cachexia models, species richness and diversity were significantly decreased with respect to the control mice. Moreover, as a concern composition, a number of taxa were found differentially represented between cachectic and non-cachectic mice, with a decrease in *Clostridia*/*Clostridiales, Lactobacillaceae*/*Lactobacillus* and an increase in *Bacteroidetes*, *Parabacteroides*, *Enterobacteriales*/*Enterobacteriaceae* which were shared by the two cachexia models [35]. In order to further investigate the changes in microbiota composition observed in cachectic mice, the authors found out that, in C26 colorectal cancer mice, the *Enterobacteriaceae* overgrowth took place above all at the expense of the butyrate-producing families of *Ruminococcaceae* and *Lachnospiraceae* and that the major contribution to *Enterobacteriaceae* bloom was provided by *Klebsiella oxytoca* [36]. This bacterial species, when exogenously administered in drinking water, was shown to act as a pathobiont contributing to gut barrier dysfunction only in cachectic but not in healthy mice and not affecting other features of cancer cachexia (such as body weight or muscle loss) [36]. Consistently with the decrease in *Ruminococcaceae* and *Lachnospiraceae* [36], when SCFAs were measured in the caecal content of C26 mice, a drop in butyrate (together with acetate) was observed, which potentially contributed to the impaired intestinal barrier [29]. A subsequent study on C26 cachectic mice confirmed the significant decrease in *Lachnospiraceae* and also described an enrichment in *Eubacteriaceae* compared to the control mice [37]. This study also revealed an altered bile acid (BA) metabolism in cachectic mice, with an increase in hepatic BAs conjugation and a decrease in the intestinal microbial production of secondary BAs. Interestingly, the levels of intestinal BAs were found to strongly correlate with *Enterobacteriaceae* but also with several species belonging to *Lachnospiraceae,* thus suggesting a marked contribution of microbiota to BAs dysregulation in cachexia [37]. An altered BA metabolism was also described in a murine model of human neuroblastoma developing cachexia symptoms, in which the microbial-produced secondary BAs lithocholic acid and deoxycholic acid were decreased in the stool samples. No significant change in microbiota composition, however, was recorded in this study, apart from a downtrend in *Firmicutes* abundance when obtained in cachectic mice [38]. The fecal microbiota was also investigated in a Lewis lung carcinoma mouse model [39], which is a recognized model of cancer cachexia [40,41]. Unlike what was previously reported [35], when compared to that of the healthy controls, the microbiota richness of cachectic lung cancer mice has significantly increased at all taxonomic levels from the order to species [39]. In addition, a LEfSe analysis of microbiota composition revealed several differences in bacterial abundance between the two groups. An overall increase in taxa belonging to *Firmicutes,* including the classes of *Bacilli* and *Clostridia* and their relative families of *Staphylococcaceae*, *Turicibacteraceae*, *Rumicococcaceae,* and *Lachnospiraceae* was observed in cachectic mice with respect to the controls. Similarly, members of *Cyanobacteria*, *Tenericutes,* and *TM7* were more abundant in cachexia. On the contrary, among *Bacteroidetes*, the families of *Prevotellaceae* and *Bacteroidaceae* and the species *Bacteroides acidifaciens* were decreased in cachectic compared to healthy mice. Among *Proteobacteria*, instead, an expansion of *Betaproteobacteria* and *Deltaproteobacteria* alongside a general decrease in *Gammaproteobacteria,* with the exception of *Enterobacteriaceae* and specifically *Escherichia coli,* was observed in cachectic mice compared to the controls [39]. Overall, several changes were found in lung cancer cachectic mice (e.g., the increase in *Lachnospiraceae* and *Ruminococcaceae* and the increase in microbial richness) with a few exceptions (e.g., the expansion in *Enterobacteriaceae*) [39], which were conflicting with previous observations in leukemic and colon cancer cachexia models [35,36,37]. As the authors suggest, differences in the mouse strains employed as well as in the source sample of microbiota (stool versus caecal content), might have contributed to such discrepancies [39]. 

In addition to bacterial dysbiosis, alterations in the intestinal mycobiota, namely the fungal community inhabiting the gut, have also been described in Lewis lung carcinoma-induced cachexia [42]. Despite no difference in alpha diversity, the stool samples from cachectic and the control mice showed enough differences in mycobiota composition to be discriminated for beta diversity. As emerged from LEfSe analysis, the *Mucoromycota* phylum was under-represented in cachectic versus control animals. The phyla of *Ascomycota* and *Basidiomycota* were overall unchanged, although a certain number of lower taxa were differentially represented between the two groups, with members belonging to *Ascomycota* enriched in cachectic mice [42].

### 3.3. Cachexia-Related Microbiota Profiles in Humans

The studies reviewed above provided some interesting insights into the relationship between gut microbiota and cancer cachexia in preclinical models. To the best of our knowledge, however, only three studies so far [28,30,43] have addressed the question of whether gut microbiota may be involved somehow in the pathogenesis of cancer cachexia in human patients. Ubachs and colleagues collected 107 patients with pancreatic (n = 27), breast (n = 52), lung (n = 24), or ovarian (n = 4) cancer and divided them into cachectic (n = 33) and non-cachectic (n = 74). The occurrence of cachexia was different, according to cancer type, with the highest in pancreatic (66.7%), followed by ovarian (25%), lung (20.8%), and breast (17.3%) cancer. Moreover, 76 healthy controls were included in the study [30]. Although no significant difference in richness and diversity metrics emerged when cachectic patients were compared to non-cachectic or to healthy controls, several differences in composition were observed. At the phylum level, *Proteobacteria* were significantly enriched in cachectic patients compared to the two other groups [30]. At the genus level, an unknown genus belonging to *Enterobacteriaceae* and *Veillonella* were significantly more abundant in cachectic subjects compared to non-cachectic and healthy people. On the contrary, *Megamonas* and *Peptococcus* were less represented among the cachectic patients than in non-cachectic and healthy controls [30]. Interestingly, when the correlation analyses of these microbial taxa with patients’ clinical variables were performed, *Veillonella* and the unknown genus of *Enterobacteriaceae* were shown to strongly correlate with body weight loss and, together with *Peptococcus*, were also positively associated with the fecal levels of calprotectin. Moreover, the unknown genus of *Enterobacteriaceae* and *Peptococcus* were also inversely correlated with the fecal levels of acetic acid, which were significantly decreased in cachectic versus non-cachectic subjects. These findings support the role of the above-mentioned bacteria in some typical manifestations of cachexia, such as weight loss and intestinal inflammation [30]. Despite being a very informative and attractive study, the heterogeneity of the patient cohort that was enrolled might represent a limitation. Indeed, since the prevalence of cachexia varies with the type of cancer, the highest contribution to the cachexia group was given by pancreatic cancer, whereas the non-cachexia group was mainly composed of breast cancer patients, so the cancer type itself should have impacted the observed results. On the other side, the studies by Ni et al. [28] and Hakozaki et al. [43] focused on lung cancer-induced cachexia. The former study recruited 12 cachectic and 19 non-cachectic lung cancer patients whose gut microbiota underwent shotgun metagenomics sequencing. Microbial richness and alpha-diversity indices were unchanged, whereas a beta-diversity analysis revealed a significantly different microbial composition between the two groups. In spite of no difference observed at the phylum level, a number of species were instead differentially represented, with the majority of the species enriched in non-cachectic patients. Interestingly, *Klebsiella oxytoca*, which was previously found to be overabundant in cachectic C26 colorectal cancer mice [36], was confirmed to be enriched in cachectic lung cancer patients [28]. Among the species under-represented in the cachexia group, instead, worth of note were *Faecalibacterium prausnitzii*, *Prevotella copri*, *Lactobacillus gasseri,* and other *Lactobacillus* species (i.e., *L. rhamnosus*, *L. plantarum*, *L. ruminis*). The changes in these microbial species seem to be correlated with the cachexia phenotype, since *F. prausnitzii* is known to be anti-inflammatory and to enhance intestinal barrier function; *P. copri* was found to be directly associated with plasma levels of isoleucine and *L. gasseri* with plasma levels of 3-oxocholic acid, while two metabolites decreased in cachectic patients [28]. Consistently, *P. copri* had been previously identified as the species that contribute the most to the biosynthesis of BCAA [44]. In addition, to uncover the taxonomic composition, the use of a shotgun metagenomic approach also allowed for the performance of functional characterization of gut microbiota, which revealed alterations in a number of pathways involved in cachexia manifestations. Among other alterations worthy of note are: (i) a decrease in the biosynthetic pathways of several amino acids, which was in agreement with the decreased plasma levels of the majority of amino acids found in cachectic lung cancer patients [28]; (ii) an increase in purine metabolism, which had been previously described in skeletal muscle atrophy [45,46]; (iii) an increase in the metabolic pathways of methane, which is reported to reduce appetite by stimulating the secretion of the anorexigenic hormone glucagon-like peptide-1 (GLP-1) [47]; (iv) an increase in the biosynthesis of lipopolysaccharide, a pro-inflammatory bacterial constituent impairing the gut barrier function [48]. The latter study on lung cancer-related cachexia confirmed previous findings that no significant difference existed in alpha-diversity metrics between cachectic and non-cachectic patients, whereas a significant clustering of the two groups was observed concerning beta-diversity. As regards composition, a number of taxa resulted in differential abundance between the two groups according to LefSe analysis. Among these, the genus *Escherichia-Shigella* (belonging to *Proteobacteria*) was enriched in cachectic patients, together with the *Christensellaceae* R-7 group, *Ruminococcaceae* UBA1819, *Lactobacillus*, *Hungatella*, *Enterococcus*, *Butyricimonas*, *Sellimonas*, *Eisenbergiella*, *Cellulosilyticum*, *Clostridium innocuum* group, *Anaerotruncus*, *Pyramidobacter*, *Ruminiclostridium, Lachnospiraceae* UCG-009, and *Paludicola*. On the contrary, in non-cachectic patients, beneficial microbes such as *Blautia*, *Roseburia*, *Eubacterium hallii* group, *Eubacterium ventriosum* group, and *Butyricicoccus* were enriched, together with *Agathobacter*, *Anaerostipes*, *Collinsella*, *Fusicatenibacter*, *Dorea*, *Megasphera*, *Monoglobus*, *Lachnospiraceae* UCG-004, and *Tyzzerella* [43].

Altogether, the findings described above strongly support that gut microbiota may play a role in the pathogenesis and clinical manifestations of cancer cachexia, which deserves further investigation.

## 4. Targeting Gut Microbiota to Manage Cancer Cachexia

**Figure 2:**
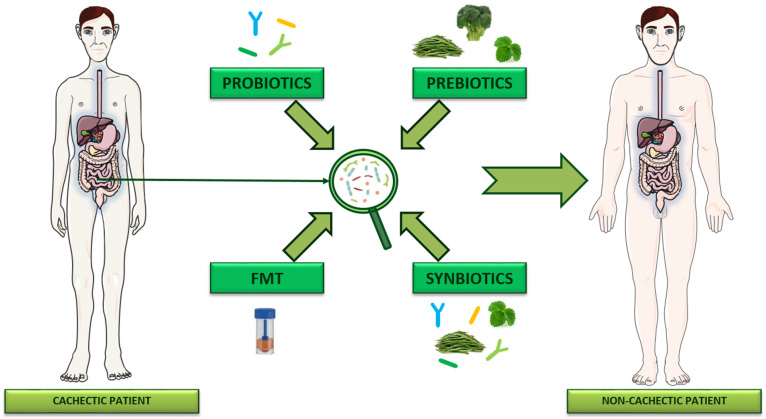
Emerging strategies of gut microbiota manipulation for the supportive treatment of cancer cachexia. Since a certain number of studies showed associations between gut microbiota and cachexia, strategies of microbiota manipulation through the administration of probiotics, prebiotics, synbiotics, or through fecal microbiota transplantation (FMT) from healthy donors are emerging to correct dysbiosis and likely improve cachexia outcome.

The studies reviewed herein, by showing associations between intestinal microbiota and the development of cachexia, suggest that manipulating gut microbiota with the aim of correcting cachexia-related dysbiosis could lead to the development of novel integrative approaches for the better management of this debilitating syndrome (Figure 2).

Few studies have already addressed this topic, showing that microbiota manipulation can positively influence the course of this multifactorial syndrome [32,34,35,42,49,50,51,52,53]. Among the available strategies for gut microbiota reshaping, live microorganisms, known as probiotics, hold a fundamental position in terms of their benefit to the human body [54]. The most important beneficial effect of probiotics is the ability to maintain the microbiota in a condition of eubiosis, to prevent infections from pathogens, to stabilize and preserve the functionality of the intestinal barrier, and to promote the production of anticancer and bioactive compounds such as various vitamins and SCFAs [55,56]. The therapeutic efficacy of probiotics depends on the fact that they can reach the intestine alive while maintaining a certain concentration (at least 10^7^ CFU per gram or mL), considering that their survival rate could decrease after oral administration due to acidic gastric pH, enzymes, bile salts, etc. [57,58]. To be defined as such, probiotics must also be able to proliferate and/or adhere to the site of action, be resistant to stress factors induced by the host organism, and be safe in use [59]. Prebiotics are also among the available intervention strategies for intestinal microbiota modulation. These non-digestible organic substances act on the selective stimulation of the growth and activity of intestinal bacteria that are beneficial for the host and his health [60,61]. Probiotics and prebiotics can work together to promote the growth of beneficial bacteria and the inhibition of pathogenic ones. This kind of intervention is named “synbiotics” and can be classified into two types: synergistic synbiotics, in which the prebiotic fibers are meant to fuel the growth of probiotic bacteria within the formulation, and complementary synbiotics, in which the two components work independently, with prebiotics aimed at promoting the growth of resident intestinal bacteria [62]. Finally, an emerging approach in the field of microbiota manipulation is fecal microbiota transplantation (FMT), which is currently proposed as a strategy to promote the restoration of a normal microbial community through the donation of a “healthy” microbiota in a dysbiotic recipient [63]. However, it must be considered that the intestinal microbiota can change its “healthy” composition in relation to the lifestyle that the individual leads and also on the basis of intestinal microbial differences that are present in different populations [64]. Furthermore, another fundamental aspect of the FMT application is the consideration of any risks that the donor incurs; for this reason, it is always necessary to carefully select donors in order to avoid the transmission of pathogens and infectious agents and expose the recipient to further risks for his health [65,66]. One of the risks for FMT recipients could be represented by the development of allergic reactions against allergens found in the donor’s fecal matter. For instance, it was observed that germ-free mice receiving FMT from children intolerant to cow milk became sensitized to milk in their turn [67]. Some authors suggest that particular attention should be paid when performing FMT in immunocompromised patients since they might be at an increased risk of developing infections compared to immunocompetent subjects [68]. On the other hand, immunocompromised patients are more prone to develop *Clostridioides difficile* infection (CDI) [69,70], which is the major indication for FMT intervention, and different studies until now have shown that FMT procedure in such immunocompromised patients is efficacy and generally safe [69,70,71]. Anyway, in order to improve both the efficacy and safety of FMT, further efforts are needed to reach a standardization of this medical intervention, as exhaustively discussed elsewhere [72,73]. 

### 4.1. Probiotics

In recent years, numerous studies have emerged in the scientific literature concerning the use of probiotics in the treatment of cancer cachexia. The most studied probiotic microorganisms were *Lactobacilli*. In particular, for the first time in this field, Bindels and colleagues reported the potential use of the genus *Lactobacillus* as a new therapeutic approach in the treatment of some aspects of cancer cachexia [34]. In this study, since in a leukemic mouse model of cachexia levels of *L. reuteri* and *L. johnsonii /gasseri* were decreased, a mix of *L. reuteri* 100-23 and *L. gasseri* 311476 was orally administered to restore their level in the intestinal tract. This intervention resulted in a reduced expression of muscular atrophy markers (Atrogin-1, MuRF1, LC3, Cathepsin L) and the production of systemic inflammatory cytokines (i.e., IL-4, Mcp-1, G-CSF, IL-6). However, it was assumed that the positive effects obtained could be species-specific since, in the same study, these effects were not demonstrated for *Lactobacillus acidophilus* [34]. Similarly, Varian and colleagues orally administered *L. reuteri* to a cachectic mouse model of colorectal cancer (C57BL/6 Apc^MIN/+^ mice; CD-1 mice) and confirmed an increase in muscle mass and a reduction in muscle atrophy compared to mice that did not receive this probiotic. They also showed an increase in lifespan, an increase in the size of the thymus., and a reduction in the expression of the FoxN1 gene associated with systemic inflammation and the production of T lymphocytes [53]. Bindels et al. also tried to counteract alterations in gut barrier function and dysbiosis observed in cachectic C26 colorectal cancer mice by administering *F. prausnitzii* [74], which was previously shown to have anti-inflammatory [75] and gut barrier-enhancing properties in colitis mice [76]. Unfortunately, this approach failed to ameliorate cachexia-related gut permeability [74]. Also, as mentioned above, the intestinal fungal population might have a role in the development of cancer cachexia [42]. Since a reduction in *Mucoromycota* was observed in cachectic Lewis lung cancer mice, the authors suggest that *Rhyzopus oryzae* (belonging to *Mucoromycota*) could be supportive in the treatment of cancer cachexia, provided that it has been recognized as safe by the Food and Drug Administration [42]. 

### 4.2. Prebiotics

In the treatment of mouse cancer cachexia, the targeted supplement of prebiotics has provided remarkable results. For example, in cachectic BaF leukemic mice, the integration of 5% polyoligosaccharides (POS) within a two-week diet led to several changes in gut microbiota composition, including a decrease in *Firmicutes* and a concomitant increase in *Bacteroidetes*, specifically in the families of *Prevotellaceae* and *Bacteroidaceae*; on the other hand, the families of *Ruminococcaceae* and *S24-7* were under-represented. At the genus level, POS induced a strong increase in *Bacteroides* (mainly due to the species *B. dorei*) in *Bifidobacterium* spp. (including *B. animalis*) and in *Roseburia* spp. POS administration also altered the SCFAs profile, inducing an increase in the caecal content of acetate and a decrease in isovalerate and branched-chain SCFAs. As a concern, metabolic manifestations and POS administration decreased anorexia and fat mass loss induced by cachexia [50]. In the same study, the effects of inulin (INU) on the cancer cachexia–related phenotype were also investigated. Regarding microbiota, a decrease in *Alistipes* spp. was observed. POS and also INU administration lowered the caecal levels of isovalerate and branched-chain SCFAs; moreover, an increase in propionate and butyrate in the portal blood was observed. A minor effect on food intake but no difference in the fat mass loss was also recorded. Finally, INU limited tumor cell invasion in the liver [50]. To modulate the development of cachexia in an Apc^MIN/+^ mouse model of colorectal cancer, Huang et al. evaluated the effect of the oral administration of ginsenoside-Rb3 and ginsenoside-Rd (two triterpene saponins) on microbiota, on the intestinal epithelium and on the production of inflammatory markers [51]. These two prebiotics, isolated from *Gynostemma pentaphyllum*, were chosen because they were previously shown to decrease tumor growth, reduce intestinal inflammation and affect gut microbiota composition in the same animal model [77]. The administration of both compounds reduced the number and size of polyps, improved the gut barrier by restoring the dysregulated expression of two transmembrane adhesion glycoproteins (E-cadherin and N-cadherin), and decreased the expression of pro-inflammatory while increasing that of anti-inflammatory cytokines. These effects were mediated by a decrement in *Proteobacteria* and in cachexia-associated microbes such as *Dysgonomonas* spp. and *Helicobacter* spp. and a concomitant increment in beneficial bacteria including *Lactobacillus* spp., *Bifidobacterium* spp., *Bacteroides acidifaciens,* and *Bacteroides xylanisolvens* [51]. More recently, the effects of supplementing cachectic mice affected by neuroblastoma with OMNi-LOGiC^®^ FIBRE (a prebiotic formula consisting mainly of dextrin and partially hydrolyzed guar gum) were investigated [78]. Compared to non-treated neuroblastoma mice, animals supplemented with prebiotics showed enrichment in the *Clostridium* family XIII-AD3011 group and *Bilophila* and a reduction in *Muribaculum*. No significant improvement in cachexia-related gut permeability was, however, seen [78]. Partially hydrolyzed guar gum was administered, in another study, to C26 cachectic mice [52], in which it counteracted the body weight loss, lowered the muscular expression of atrophy (i.e., Atrogin-1, MuRF1) and autophagy markers (Lc3a, Lc3b, and FoxO1), thickened the intestinal mucus layer, and decreased the circulating levels of IL-6. Moreover, this prebiotic also induced changes in mice gut microbiota, reducing alpha-diversity, promoting *Firmicutes* growth at the expense of *Bacteroidetes*, and decreasing *Proteobacteria* and *Oscillospira* while increasing *Bifidobacterium* and *Akkermansia* [52].

### 4.3. Synbiotics

As described above, since a decrease in *Lactobacillus* spp. was observed in cachectic C26 and Ba/F3 mice compared to the controls, Bindels et al. set up a synbiotic formulation containing *L. reuteri* 100-23 and inulin-type fructans which were administered to leukemic mice. This approach, though not affecting the animals’ energy intake, significantly counteracted muscle mass atrophy, increased caecal mass and content, and affected the gut microbiota composition by restoring many of the taxa associated with cachexia, such as decreasing *Escherichia* and *Anaerotruncus* and enriching *Lactobacillus*, *Bifidobacterium*, *Barnesiella,* and *Parasutterella.* These microbial alterations were associated with improvements in the intestinal barrier and immunological functions [35]. Another study investigated, in C26 mice, the cachexia-preventive action of kimchi: a Korean fermented product containing mixed vegetables and the probiotic strains *Leuconostoc mesenteroides* CJ LM119 and *Lactobacillus plantarum* CJ LP133 [49]. Animals receiving kimchi showed a significant reduction in body weight loss, remarkable preservation of muscle mass, and decreased expression of atrophy markers, despite no change in food intake and tumor size. Moreover, a reduction in both muscular and serum levels of IL-6, together with significant inhibition of the IL-6 downstream inflammatory pathway, compared to non-treated counterparts was observed. Finally, a lipolysis-preventive effect was also obtained [49].

### 4.4. Fecal Microbiota Transplantation

Nowadays, the only approved clinical indication for FMT is represented by CDI, for which new formulations (i.e., orally administrable) are being launched with promising results [79]. On the contrary, FMT application for the treatment of other diseases is still in an exploratory phase. To the best of our knowledge, only two reports of FMT as a corrective approach for cancer cachexia exist. In the first study, the employment of germ-free mice compared to conventional ones demonstrated the importance of intestinal microbiota in regulating skeletal muscle mass and function. When gut microbiota was restored in germ-free mice through FMT from conventional animals, muscle mass was recovered, muscle atrophy markers were lowered, muscular energy and protein metabolism were re-established, and locomotor activity and neuromuscular junctions returned to normal [32]. The study by de Clercq et al. represents the only report of microbiota manipulation on human cachectic patients. In detail, autologous or allogenic FMT from obese donors was performed on 24 cachectic patients with metastatic gastroesophageal cancer. The engraftment of the FMT was confirmed by analyzing gut microbiota before and after the transplantation in both groups since, although not showing differences in alpha-diversity metrics, the taxonomic composition of patients after receiving allogenic FMT was different from the baseline and similar to that of the donors. This shift did not occur in patients receiving autologous FMT. Improvements in the disease control rate, overall survival, and progression-free survival were observed in patients receiving allogenic FMT; nevertheless, no significant positive effect on cachexia outcome was recorded, as demonstrated by unaffected appetite and caloric intake [80].

## 5. Conclusions

Cancer cachexia is a serious but still neglected medical issue for which no effective and resolutive treatment has been so far identified, so patients’ quality of life, clinical outcome, and overall survival remain negatively compromised. As reviewed in this study, several lines of evidence show a potential link between gut microbiota with their metabolites, and cancer cachexia symptoms and markers, thus suggesting that manipulation of microbiota could help in restoring eubiosis and relieving the clinical manifestations of this syndrome. Among the strategies for reshaping gut microbiota, the administration of probiotics, prebiotics, and synbiotics provided some encouraging, albeit preliminary, results. In addition, the FMT is arising as a way to re-establish a health-associated microbiota in a number of disease conditions, including cancer cachexia which, however, is still in its infancy.

Due to the novelty of these microbiota manipulation approaches, the efficacy and safety of long-term effects remain unexplored. Moreover, almost all the evidence gathered so far associates microbiota strategies of manipulations and cancer cachexia come with preclinical models, whereas human studies are still lacking. For these reasons, further investigations are needed to fully exploit the potential of microbiota-targeted interventions in the clinical management of cancer cachexia.

## Data Availability

Not applicable.
